# Cross-cultural adaptation and validation of the Italian version of the Western Ontario Osteoarthritis of the Shoulder index (WOOS)

**DOI:** 10.1007/s10195-016-0400-4

**Published:** 2016-03-31

**Authors:** Katia Corona, Simone Cerciello, Brent Joseph Morris, Enrico Visonà, Giovanni Merolla, Giuseppe Porcellini

**Affiliations:** 1 Science for Health Department, Molise University, Via De Sanctis, 86100 Campobasso, Italy; 2 Catholic University of Rome, Largo Francesco Vito 1, 00168 Rome, Italy; 3 Lexington Clinic Orthopedics-Sports Medicine Center, The Shoulder Center of Kentucky, Lexington, USA; 4 Ospedali Riuniti Padova Sud-ULSS 17, Schiavonia (PD), Italy; 5 Unit of Shoulder and Elbow Surgery, D. Cervesi Hospital, Cattolica-AUSL della Romagna Ambito Territoriale di Rimini, Cattolica, Italy

**Keywords:** Shoulder osteoarthritis, Western Ontario Osteoarthritis of the Shoulder index, Cross-cultural adaptation, Validation

## Abstract

**Background:**

The Western Ontario Osteoarthritis of the Shoulder index (WOOS) has been introduced as a disease-specific quality of life measurement in patients with glenohumeral arthritis. The aim of the present study was to perform a cross-cultural adaptation of the English version of the WOOS to Italian and to assess its validity, reliability and responsiveness in patients with glenohumeral joint osteoarthritis treated conservatively.

**Material and methods:**

The adaptation process was carried out following the simplified Guillemin criteria. The English version was translated into Italian by two bilingual orthopaedic surgeons and then translated back into English by two different bilingual orthopaedic surgeons. The original version was compared with the back-translation. The questionnaire was prospectively administered to 30 patients with glenohumeral osteoarthritis at baseline and again after 5 days for retest reliability. After 6 months of conservative treatment, the responsiveness of the questionnaire was assessed in a subsample of 20 patients. The level of statistical significance was set at 0.05.

**Results:**

The interclass correlation coefficient between test and retest of the WOOS was 0.99 (*P* < 0.001). Pearson’s correlation coefficient between the WOOS and disability of the arm, shoulder and hand (DASH) preoperatively was 0.73 (*P* < 0.01) and the correlation between the changes of score for the WOOS and DASH was 0.75 (*P* < 0.01). There were no floor or ceiling effects. Responsiveness, calculated by standardized response mean, was 1.1 and effect size was 1.3.

**Conclusions:**

The Italian version of the WOOS questionnaire has shown to be equivalent to its English version and demonstrated good validity, reliability and responsiveness to conservative treatment of glenohumeral osteoarthritis.

**Level of evidence:**

Level II.

**Electronic supplementary material:**

The online version of this article (doi:10.1007/s10195-016-0400-4) contains supplementary material, which is available to authorized users.

## Introduction

Patient-reported outcomes are used to assess the severity of pathology and evaluate the outcomes of both conservative and surgical treatments. Most of these questionnaires are created and developed in English-speaking regions and tested on the cultural traditions in these areas. A simple translation of these scoring systems into different languages and cultures is not sufficient. These outcome tools must be validated with a process of translation and adaptation before being used in practice [1, 2]. This process is not a mere translation and must take into account language and cultural differences. Guillemin was the first to describe this process consisting of translation of the questionnaire and subsequent adaptation to idioms, culture and lifestyle. He described a 5-step process including translations and back-translations by qualified people, a committee review of these translations and back-translations, pre-testing for equivalence, and finally a re-examination of the weighting of scores. These aspects are important in current practice since most of these questionnaires are created in English-speaking countries where quality of life or expectancies and subjective assessment may be different from the countries where they are later introduced. At the end of this process, a statistical evaluation including validity, reliability and responsiveness to treatment (conservative or surgical) must be carried out before considering these scoring systems suitable to be used in different countries [3]. The aim of the present study was to perform a cross-cultural adaptation and validation of the Western Ontario Osteoarthritis of the Shoulder (WOOS) index into Italian and to assess its reliability.

## Materials and methods

### Outcome tools

#### Western Ontario Osteoarthritis of the Shoulder index

The WOOS index is a patient-administrated, disease-specific questionnaire for measurement of the quality of life of patients with osteoarthritis of the shoulder [4]. It investigates four domains of the patient’s life: physical symptoms, sport/recreation/work, lifestyle and emotions. Nineteen questions are specific to these aspects and the answer is given on a visual analogue scale with a possible score ranging from 0 to 100. Therefore, a score of 1,900 indicates that the quality of life is extremely affected by the shoulder, whereas a score of 0 signifies that the patient has no decrease in their shoulder-related quality of life. A forward translation of the WOOS from English to Italian was carried out by two independent physicians. An accurate comparison of these two translated Italian versions was performed to create a new single one. A backward translation from Italian to English was then performed by two other physicians and checked for inconsistencies with the original English text. No additional adaptations were performed regarding cultural differences between English-speaking regions and Italy.

The final version of the questionnaire was then administered to a selected population. The three aspects mentioned, validity, reliability and responsiveness, were investigated. Validity represents the meaningfulness, appropriateness and utility of a measurement. Reliability is the ability to provide the same result in stable subjects and adequate levels of measurement variability with repeated administration of a measurement tool. Finally, the responsiveness is the ability of a questionnaire to reflect significant clinical change in the subject’s state after treatment [5]. Floor and ceiling effects were also evaluated. The floor effect occurs when an individual scores at the bottom of a scale and no further decline can be registered. Ceiling effects occur at the top of a scale so that no further improvement can be registered. These aspects were assessed by comparing the WOOS and the Italian validated version of the disability of the arm, shoulder and hand score (DASH). The DASH was administered to the same study population and was then compared to the Italian version of the WOOS [6].

### Patients

Thirty-two patients (2 male, 30 female) affected by glenohumeral joint osteoarthritis were prospectively evaluated for enrolment in the present study. Each patient was required to be a candidate for conservative treatment of early stage glenohumeral joint osteoarthritis in order to be included. Two patients with fibromyalgia were excluded at the time of enrolment, since the diagnosis may have affected patient perception of the local pathology. Thirty patients were deemed eligible and enrolled in the study (1 male, 29 females). Mean age at the time of first evaluation was 65 years (range 62–73 years). All patients were assessed with physical examination and standard radiographic evaluation consisting of true anterior–posterior views of the shoulder with the arm in internal, neutral and external rotation. The diagnosis of glenohumeral joint osteoarthritis was confirmed radiographically in all patients (stage 1 in 21 patients and stage 2 in 9 patients according to the classification introduced by Samilson and Prieto). All patients were asked to complete the WOOS and DASH questionnaires in the presence of an orthopaedic resident. The time necessary to complete each one of the questionnaires and any difficulty encountered in answering a question was recorded. To reduce the risk of short-term clinical change, no treatment was provided to these patients over a 5-day interval. To perform test–retest evaluation and test the reliability of the questionnaire, patients were asked to complete the same questionnaires 5 days later, assuming that the clinical situation and severity of symptoms had not changed during this short interval. Twenty patients agreed to undergo a protocol of conservative treatment consisting of stretching exercises, strengthening and active exercises over a period of 6 months. At the end of the program, the same score sheets were administered to these patients. This allowed calculation of the responsiveness of the questionnaire. In addition, the distribution of scores and the ceiling and floor effects were calculated by examining the item responses.

### Statistical analysis

**Fig. 1:**
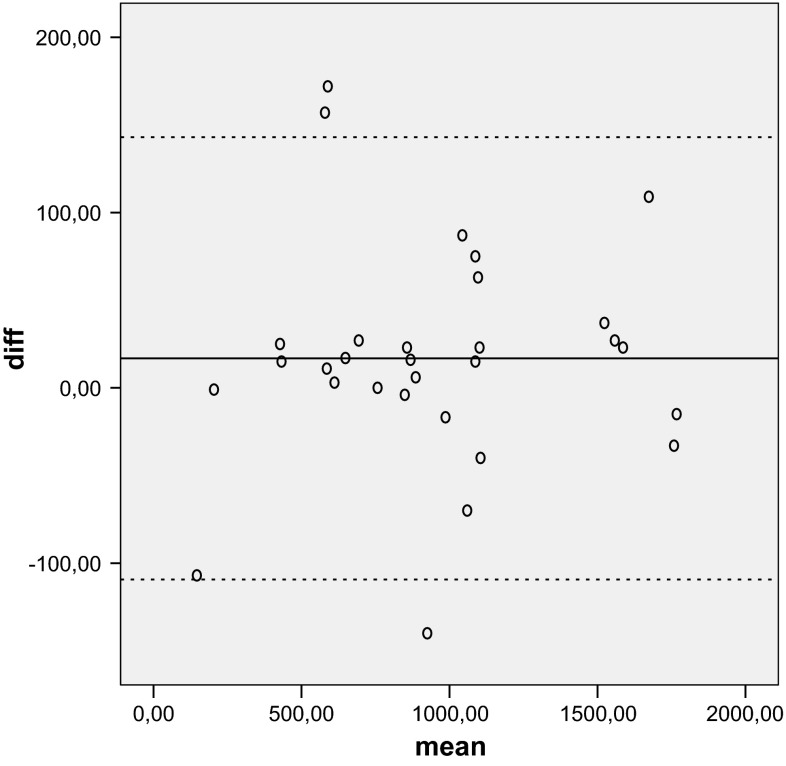
Bland–Altman plot shows the test and retest results for 30 patients completing the Italian version of the Western Ontario Osteoarthritis of the Shoulder (WOOS) index. The *solid line* shows the mean difference and the *dashed lines* show the upper and lower 95 % confidence intervals

Statistical analysis was performed using SPSS 11.5 for Windows. The Shapiro–Wilk test was used to assess normality. Correlation between WOOS and DASH was assessed with a parametric test (Pearson’s correlation) and the test–retest reliability was assessed with interclass correlation coefficient (ICC) for the total score and for the four domains. Absolute reliability was determined by estimating the standard error of measurement SEM = SD × √(1 − ICC), where SD is the standard deviation, and the minimum detectable difference MDD = 1.96 × √2 × SEM. A Bland–Altman plot shows the mean difference in test and retest values of WOOS against the mean of these two measures (Fig. 1). Responsiveness was assessed by the standardized response mean (SRM) and the effect size (ES). SRM is calculated as the difference between the preoperative mean score and the postoperative mean score divided by the SD of the difference. ES is calculated as the difference between the postoperative mean score and the preoperative mean score divided by the preoperative SD.

Ceiling and floor effects were investigated since they also have an effect on the responsiveness of a measure. All tests were two-sided, and values of *P* < 0.05 were considered to be statistically significant.

## Results

### Validity

A correlation was performed to assess the construct validity between WOOS and DASH. Cronbach’s alpha was 0.910. The initial Pearson’s correlation coefficient between the WOOS and DASH was 0.73 (*P* < 0.01), and the correlation between the score at the end of the conservative treatment was 0.75 (*P* < 0.01) (Table 1). The correlation was strong and equivalent to the results presented for the original English version. reported as 0.73 and 0.69, respectively [4]. There were no floor or ceiling effects preoperatively or postoperatively for the total WOOS.

**Table 1:** Intraclass correlation coefficients (*ICC*) of the four domains and the total Western Ontario Osteoarthritis of the Shoulder (WOOS) index (*n* = 30)

WOOS domains	ICC*
Physical symptoms	0.98
Sport/recreation/work	0.99
Lifestyle	0.98
Emotions	0.99
Total WOOS score	0.99

* Values for *P* for the ICCs were all <0.001

### Test–retest reliability

The mean WOOS was calculated at initial evaluation and over a 5-day interval. Values were 925 and 919, respectively. The ICC for the total WOOS was 0.99, and for the domains physical symptoms 0.98, sport/recreation/work 0.99, lifestyle 0.98 and emotion 0.99. All values were highly statistical significant (*P* < 0.001) (Table 2). The test–retest reliability of the WOOS was high, with an excellent ICC for the domains and for the total score, superior to the results presented in the original English version, which reported an ICC of the domains between 0.87 and 0.95 and total ICC value of 0.96 [4]. The SEM/MDC was 0.80/2.22 for WOOS, indicating a smaller amount of measurement error in the screen. A Bland–Altman plot showed a small mean difference.

**Table 2:** Correlation between measures

	DASH	WOOS
DASH	1	0.73**
WOOS	0.73**	1

*Note* Pearson’s correlation coefficient between the WOOS and DASH

*WOOS* Western Ontario Osteoarthritis of the Shoulder index, *DASH* disability of the arm, shoulder and hand score

** Correlation is significant at the 0.01 level (2-tailed)

### Responsiveness

The WOOS was responsive and sensitive to detecting clinical changes in the study population after a 6-month period of conservative treatment. The SRM for the domains of WOOS ranged from 0.8 to 1.3. The SRM for the total WOOS was 1.1 and for the total DASH was 0.9 (Table 3). The result was very positive, since a SRM >0.8 is generally considered to be excellent. Ceiling and floor effects, which also have an effect on the responsiveness of a measure, were absent. In fact in the present study, no patient rated “no shoulder function” or “full shoulder function” using the WOOS or the DASH.

**Table 3:** Responsiveness of the WOOS and DASH (*n* = 20)

Domains	SRM	ES
Physical	0.98	1.12
Sport/recreation/work	1.30	1.42
Lifestyle	1.13	0.98
Emotions	0.81	1.05
Total WOOS score	1.11	1.33
DASH score	0.90	1.07

*Note* The SRM and ES of the four domains of the WOOS, the total WOOS and DASH

*WOOS* Western Ontario Osteoarthritis of the Shoulder index, *DASH* disability of the arm, shoulder and hand score, *SRM* standardized response mean, *ES* effect size

## Discussion

The glenohumeral joint is a common cause of chronic joint pain and only second behind the knee joint (30.6 vs. 63.4 %) [7]. Although it is the third most common large joint affected by degenerative joint disease, clinically significant osteoarthritis is relatively less frequent.

In 2004, approximately 4 % of the total joint prostheses involved the glenohumeral joint [8]. Moreover, between 1998 and 2008 there was a 2.5-fold increase (from 19,000 to 47,000) in implanted shoulder arthroplasties performed in the USA [9]. Treatment options comprise both non-operative and operative approaches, including activity modification, nonsteroidal anti-inflammatory medications, corticosteroid injections, and shoulder replacement. An accepted patient-reported disease-specific outcome tool would be of great interest when evaluating the severity of symptoms and the efficacy of these treatment options. Different scoring systems have been developed for specific conditions. One of the advantages of these tools is the ability to compare results in different countries and to facilitate cultural exchange between physicians and multi-centre studies. However, most of these scoring systems are in English and have been created for the culture of English-speaking countries. These scoring systems are not necessarily generalizable to other non-English-speaking countries. The process of creating these questionnaires in another language is not a simple translation, rather it involves a cross-cultural adaptation [2], which has been thoroughly described by Guillemin et al. [1]. At the end of process the tool can be effective for comparing results in multicentre studies with minimal biases and improved precision in meta-analyses [2, 10]. The WOOS questionnaire was introduced in 2001 to be used in patients with glenohumeral joint osteoarthritis, and showed good validity and reliability [4]. The time to administer the test is generally 10 min, and the ease of scoring has been rated as moderate [11]. It was then used in patients with subacromial pain [12] and to assess the outcomes of arthroscopic debridement in subjects with arthritis [13]. The questionnaire been has validated in different languages: English, French, Spanish, German, Swedish and Danish [12, 14, 15].

The present study aimed to adapt the WOOS questionnaire into Italian and to assess its validity and reliability. In addition, the responsiveness to conservative treatment was assessed. The WOOS strongly correlated with the DASH score, which serves as a gold standard, indicating good validity. The test/retest reliability was very high, indicating that the score was consistent over a short period of time. Finally, an additional aim of the study was to assess the influence of conservative treatment on the perception the patients had of their shoulder problem. The responsiveness to conservative treatment was good, indicating that the treatment positively influenced patient perception. The results of the present study were comparable with those of previous studies [12, 15]. However, prior studies looked at operatively treated patients, and the effect of an entire cohort of patients with glenohumeral joint osteoarthritis treated conservatively has not been tested before. In addition, the lack of floor and ceiling effects confirms the validity of this version of the aforementioned scoring scales. The ceiling effect usually happens when all testers score very high, and the floor effect occurs when most of them score very low. The presence of these effects makes data analysis difficult and prevents achieving good reliability for a test.

The present study does have some limitations. The most important one is the lack of a power analysis. To reduce the risk of potential biases, we referred to similar studies available in the literature to determine the sample size needed. One of the strengths of the study is that this is the first time the WOOS index has ever been translated into Italian and applied. Furthermore, this was a very homogeneous patient population consisting of glenohumeral joint osteoarthritis and all patients were treated conservatively with a standardized protocol.

Currently no Italian validated version of the WOOS index is available. The present study confirms that the scoring system has high correlation with the DASH score. The test–retest reliability was also high. In addition, the Italian WOOS index showed good responsiveness, indicating that it is positively influenced by conservative treatment. The scoring system also demonstrated no substantial ceiling or floor effects. The Italian version of the WOOS index can be reliably used in Italian patients with glenohumeral osteoarthritis.

## Electronic supplementary material

Below is the link to the electronic supplementary material.

Supplementary material (DOC 131 kb)
