# Supplementary material for: Cross-cultural adaptation and validation of the Italian version of the Western Ontario Osteoarthritis of the Shoulder index (WOOS)
Source: J Orthop Traumatol. 2016 Mar 31;17(4):309–13. doi: 10.1007/s10195-016-0400-4 (PMC5071231; doi:10.1007/s10195-016-0400-4)
Supplement: Supplementary file 1 — Supplementary material (DOC 131 kb) [file 10195_2016_400_MOESM1_ESM.doc]

SCHEDA DI VALUTAZIONE WESTERN ONTARIO OSTEOARTHRITIS OF THE SHOULDER (WOOS)

ISTRUZIONI PER I PAZIENTI

Nel questionario vi verrà chiesto di rispondere a delle domande secondo il seguente formato e dovrete rispondere barrando con il seguente segno “/” la linea orizzontale.

NOTA BENE:

1. Se ponete il segno “/” all’estremità sinistra della linea come segue:


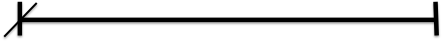


state indicando che non siete affetti da questa condizione in nessun modo

2. Se ponete il segno “/” all’estremità destra della linea come segue:


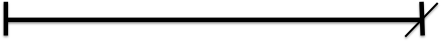


state indicando che siete affetti da questa condizione in modo estremo

3. Tenete inoltre presente che:

a) Più ponete a destra il segno “/”, più indicate di essere affetti da quel sintomo.

a) Più ponete a sinistra il segno “/”, meno indicate di essere affetti da quel sintomo.

c) cortesemente non ponete il segno “/” al di fuori della linea.

Nel seguente questionario, vi verrà chiesto di indicare l’entità dei sintomi che hanno afflitto l’arto in questione nella settimana passata. Se non siete sicuri riguardo a quale spalla sia maggiormente interessata o se avete ogni genere di domanda, chiedete al medico prima di compilare il questionario. Se per qualche ragione non comprendete una domanda, potete fare riferimento alle spiegazioni menzionate alla fine del questionario. Dopo potrete porre il segno “/” sulla linea orizzontale nel posto che riterrete più opportuno. **Se un argomento non fa riferimento alla vostra condizione o se comunque non ne siete stati interessati nella settimana precedente, vi preghiamo di tentare la stima più realistica, così da rendere la risposta accurata.**

SEZIONE A: Sintomi clinici

ISTRUZIONI PER I PAZIENTI

**Le domande seguenti si riferiscono ai sintomi clinici che avete avuto, in relazione al problema della vostra spalla. In tutti i casi, riferite l’entità dei sintomi che avete avuto nella settimana passata. (Rispondete a ciascuna domanda con il segno “/”)**

1. A seguito dei movimenti, quanto dolore acuto avete avuto alla spalla?

nessun dolore
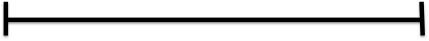
estremo dolore

1. Quali sono l’entità e la continuità del dolore che avete alla spalla?

nessun dolore
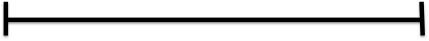
estremo dolore

1. Quanto è debole la vostra spalla?

per nulla
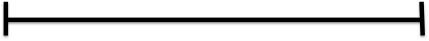
estremamente debole

1. Quanto è rigida la vostra spalla?

per nulla
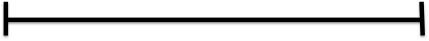
estremamente rigida

1. Quanti scatti e scrosci sentite nella vostra spalla?

nessuno
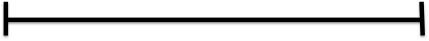
moltissimi

1. In che entità le condizioni metereologiche influenzano la vostra spalla?

per nulla
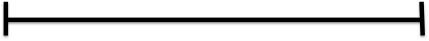
estremamente

SEZIONE B: Sport/Tempo libero/Lavoro

ISTRUZIONI PER I PAZIENTI

**Le sezione seguente si riferisce a come il problema della vostra spalla ha influenzato le vostre attività sportive, lavorative o di tempo libero nella settimana passata. Rispondete a ciascuna domanda con il segno “/”**

1. Quanta difficoltà avete nel lavorare o muovere l’arto sopra il livello della spalla?

nessuna difficoltà
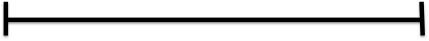
estrema difficoltà

1. Quanta difficoltà avete nel sollevare oggetti (es. sacchi della spesa, spazzatura, etc.) sotto il livello della spalla?

nessuna difficoltà
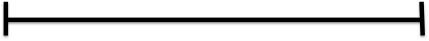
estrema difficoltà

1. Quanta difficoltà avete nell’eseguire movimenti ripetitivi sotto il livello della spalla come: rastrellare, spazzare, innaffiare i fiori a causa della vostra spalla?

nessuna difficoltà
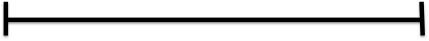
estrema difficoltà

1. Quanta difficoltà avete nello spingere o tirare qualcosa con forza a causa della vostra spalla?

nessuna difficoltà
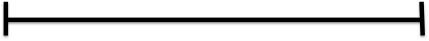
estrema difficoltà

1. Quanto siete in difficoltà a causa dell’aumento del dolore alla vostra spalla dopo le attività fisiche?

per nulla
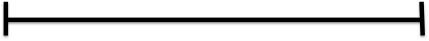
estremamente

in difficoltà

SEZIONE C: Stile di vita

ISTRUZIONI PER I PAZIENTI

**Le sezione seguente si riferisce a come il problema della vostra spalla ha influenzato il vostro stile di vita. Indicate l’entità corretta, relativamente alla settimana passata, mediante il segno “/”**

1. Quanta difficoltà avete a dormire a causa della vostra spalla?

nessuna difficoltà
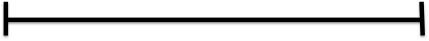
estrema difficoltà

1. Quanta difficoltà avete nel pettinarvi i capelli a causa della vostra spalla?

nessuna difficoltà
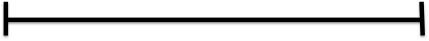
estrema difficoltà

14. Quanta difficoltà avete nel mantenere lo stato di forma desiderato a causa della vostra spalla?

nessuna difficoltà
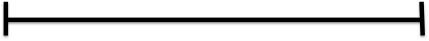
estrema difficoltà

1. Quanta difficoltà avete nel portare l’arto posteriormente per infilare una camicia, prendere il portafogli dalla tasca posteriore dei pantaloni, abbottonarvi il vestiario, a causa della vostra spalla?

nessuna difficoltà
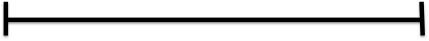
estrema difficoltà

1. Quanta difficoltà avete nel vestirvi e nello spogliarvi a causa della vostra spalla?

nessuna difficoltà
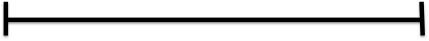
estrema difficoltà

SEZIONE D: Emozioni

ISTRUZIONI PER I PAZIENTI

**Le domande seguenti si riferiscono o come vi siete sentiti nella passata settimana in relazione alla problematica della vostra spalla. Indicate la vostra risposta mediante il segno “/”**

1. Quanta frustrazione o scoramento provate a causa della vostra spalla?

nessuna
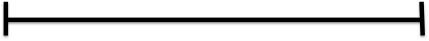
estrema

1. Quanto siete preoccupati per quello che accadrà alla vostra spalla in futuro?

nessuna
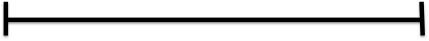
estrema preoccupazione

preoccupazione

19. Quanto vi sentite di peso per gli altri?

per nulla
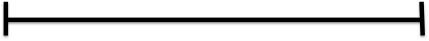
di peso estremo

GRAZIE PER AVER COMPLETATO IL QUESTIONARIO

**Spiegazione del significato delle domande del questionario WOOS**

**Sezione A: Sintomi clinici**

***Domanda 1***

Si riferisce ad ogni dolore acuto o tipo spasmo, improvviso che provate muovendo il braccio

***Domanda 2***

Si riferisce al dolore sordo, persistente, di fondo che è quasi costante in opposizione a quello improvviso menzionato nella spiegazione della domanda 1

***Domanda 3***

Si riferisce alla mancanza di forza nello svolgere movimenti o attività

***Domanda 4***

Si riferisce alla sensazione che la vostra spalla non si “voglia” muovere o che sembri congelata o bloccata. Questo può essere riscontrato la mattina dopo essersi alzati o dopo un periodo di inattività

***Domanda 5***

Si riferisce ad ogni rumore e/o sensazione che sentite nella vostra spalla a seguito di movimenti che spesso può essere descritto come: scroscio, stridore, scatto, fruscio

***Domanda 6***

Molte persone hanno la percezione che il dolore o la rigidità nella loro spalla cambia con il tempo. Alcuni lo sentono con il cambiamento della pressione o della temperatura atmosferica. Considerate pertanto tutte queste condizioni

**Sezione B: Sport/Lavoro/Tempo libero**

***Domanda 7***

Si riferisce ad ogni attività che richiede di sollevare l’arto sopra il livello della spalla. Ad esempio cambiare una lampadina da una lampada a soffitto, pulire una finestra, raggiungere uno scaffale alto, appendere i vestiti in un armadio, o piegare un lenzuolo grande

***Domanda 8***

Si riferisce a sollevare oggetti ad ogni altezza purché sotto il livello della spalla. Ad esempio portare una busta della spesa, una palla da bowling, una cassa di bibite, spazzatura, libri, attrezzature da lavoro, o mettere qualcosa nel forno.

***Domanda 9***

Si riferisce all’esecuzione di movimenti ripetitivi avanti ed indietro, o circolari sotto il livello della spalla

***Domanda 10***

Si riferisce ad ogni azione che richieda forza per la sua esecuzione come tirare o spingere un aspirapolvere pesante, aprire o chiudere una porta o una finestra pesante, portare via piante da un giardino, tirare via i calzini, cambiare le marce di una auto o muovere mobili.

***Domanda 11***

Si riferisce al dolore aggiuntivo che sentite dopo aver svolto attività che richiedono l’uso della vostra spalla

**Sezione C: Stile di vita**

***Domanda 12***

Si riferisce all’influenza che la vostra spalla ha sul vostro modo abituale di dormire, ad esempio la necessità di cambiare posizione, il fatto di svegliarsi la notte, difficoltà ad addormentarsi, difficoltà a trovare una posizione confortevole, o svegliarsi sentendosi ancora stanco.

***Domanda 13***

Si riferisce ad ogni cosa voi facciate con i vostri capelli, quale pettinarsi, spazzolarsi o lavarsi che richieda sollevare le braccia (e la spalla in questione) per arrivare ai capelli

***Domanda 14***

Si riferisce al livello dell’attività fisica che siete riusciti a mantenere da quando la vostra spalla è diventata un problema al momento attuale. Considerate tutte le attività che a vostro avviso contribuiscono al tono muscolare, alla vostra forza, all’esercizio cardio-vascolare quali, bowling, curling, passeggiare, o canottaggio, etc

***Domanda 15***

Si riferisce al fatto di riuscire a portare l’arto posteriormente per chiudere o aprire una cerniera o reggiseno, raggiungere la tasca posteriore dei pantaloni, grattarsi la schiena, etc

***Domanda 16***

Si riferisce ad ogni difficoltà nel mettersi o levarsi i vestiti, cambiare taglia o stile di abbigliamento a causa della spalla, o cambiare il modo di vestirsi o spogliarsi a causa della vostra spalla

**Sezione D: Emozioni**

***Domanda 17***

Si riferisce ad ogni frustrazione causata dalla vostra spalla. Si può intendere frustrazione per le limitazioni fisiche o situazione economica ma che siano correlate alle problematiche della vostra spalla

***Domanda 18***

Si riferisce alla paura che la vostra spalla possa peggiorare invece di migliorare o rimanere stabile

***Domanda 19***

Si riferisce alla sensazione che stiate costringendo il prossimo ad aiutarvi per le mansioni quotidiane o per cose che abitualmente facevate da soli

SISTEMA DI PUNTEGGIO PER IL QUESIONARIO WOOS

1. Misurate la distanza dal margine sinistro della linea orizzontale e calcolate il punteggio su un massimo di100 (approssimato al più vicino 0.5mm). Riportate il valore nello spazio previsto per quella domanda.
2. Si può pertanto calcolare il valore per ogni gruppo (Sintomi clinici/600, Sports/lavoro/500, Stile di vita/500, emozioni/300) o il valore totale dalla somma di tutti i gruppi su un massimo di 1900
3. Alcuni ritengono più significativo riportare i punteggi su un massimo di 100, cioè una percentuale del punteggio normale. Siccome il punteggio più basso possibile è 1900, il punteggio risultante dalla somma dei gruppi viene sottratto da 1900 e diviso per 19.

Es. punteggio totale dei gruppi = 1625; 1900 – 1625 = 275/19 = 14.5%

Sintomi clinici Sport/Lavoro/Tempo libero Stile di vita

| SC 1 |  |
| --- | --- |
| SC 2 |  |
| SC 3 |  |
| SC 4 |  |
| SC 5 |  |
| SC 6 |  |
| TOTALE |  |

| SLT 1 |  |
| --- | --- |
| SLT 2 |  |
| SLT 3 |  |
| SLT 4 |  |
| SLT 5 |  |
| TOTALE |  |

| SV 1 |  |
| --- | --- |
| SV 2 |  |
| SV 3 |  |
| SV 4 |  |
| SV 5 |  |
| TOTALE |  |

Emozioni TOTALE

| SC |  |
| --- | --- |
| SLT |  |
| SV |  |
| E |  |
| TOTALE |  |

| E 1 |  |
| --- | --- |
| E 2 |  |
| E 3 |  |
| TOTALE |  |
